# *ZnT3* Gene Deletion Reduces Colchicine-Induced Dentate Granule Cell Degeneration

**DOI:** 10.3390/ijms18102189

**Published:** 2017-10-19

**Authors:** Bo Young Choi, Dae Ki Hong, Sang Won Suh

**Affiliations:** Department of Physiology, College of Medicine, Hallym University, Chuncheon 24252, Korea; bychoi@hallym.ac.kr (B.Y.C.); zxnm01220@gmail.com (D.K.H.)

**Keywords:** *ZnT3*, colchicine, axonal transport, zinc, neuron death, oxidative stress, glutathione

## Abstract

Our previous study demonstrated that colchicine-induced dentate granule cell death is caused by blocking axonal flow and the accumulation of intracellular zinc. Zinc is concentrated in the synaptic vesicles via zinc transporter 3 (*ZnT3*), which facilitates zinc transport from the cytosol into the synaptic vesicles. The aim of the present study was to identify the role of *ZnT3* gene deletion on colchicine-induced dentate granule cell death. The present study used young (3–5 months) mice of the wild-type (WT) or the *ZnT3*^−/−^ genotype. Colchicine (10 µg/kg) was injected into the hippocampus, and then brain sections were evaluated 12 or 24 h later. Cell death was evaluated by Fluoro-Jade B; oxidative stress was analyzed by 4-hydroxy-2-nonenal; and dendritic damage was detected by microtubule-associated protein 2. Zinc accumulation was detected by *N*-(6-methoxy-8-quinolyl)-para-toluenesulfonamide (TSQ) staining. Here, we found that *ZnT3*^−/−^ reduced the number of degenerating cells after colchicine injection. The *ZnT3*^−/−^-mediated inhibition of cell death was accompanied by suppression of oxidative injury, dendritic damage and zinc accumulation. In addition, *ZnT3*^−/−^ mice showed more glutathione content than WT mice and inhibited neuronal glutathione depletion by colchicine. These findings suggest that increased neuronal glutathione by *ZnT3* gene deletion prevents colchicine-induced dentate granule cell death.

## 1. Introduction

Colchicine, a potent neurotoxin derived from plants of the genus *Colchicum autumnale*, is well known to cause selective loss of dentate granule cells in the hippocampus [1,2], and to cause cognitive dysfunction [3] resulting from cytoskeletal alterations and impaired axonal transport, followed by progressive neuronal loss. The neuronal cytoskeleton is a system of highly complex structures that consist of microtubules, neurofilaments and microfilaments. These components are responsible for the supportive shape of neurons, as well as for crucial processes such as transport of materials in the axon [4]. It has been found that colchicine injection decreases the soluble tubulin pool and inhibits microtubule polymerization by binding tightly to tubulin, the major structural protein of microtubules [5]. Kumar et al. demonstrated that intracerebral administration of colchicine induces excessive free radical generation and consequently oxidative damage [6,7]. The central nervous system (CNS) is highly vulnerable to oxidative stress because of its increased oxygen consumption for lipid peroxidation and is relatively deficient in antioxidant systems [8,9]. Therefore, generation of free radicals and oxidative stress can cause neuronal death.

Glutathione (GSH) is an intracellular non-protein thiol that plays a central role in antioxidant defense against free radical production, especially reactive oxygen species (ROS) production. Excess of ROS may result in GSH depletion [10,11,12]. The onset of cell death is associated with a reduction of intracellular GSH levels in various cellular systems [13,14]. Therefore, attenuation of oxidative stress by increasing antioxidant defense through modulation of GSH can be a useful tool in the management of neurodegeneration processes [15,16,17].

The zinc (Zn^2+^) ion, one of the most abundant trace metals in the CNS, is enriched in the human body and has been known to be important in the control of physiological and pathological functions in the brain [18,19,20,21]. Anterograde and retrograde zinc transporters between the cell body and the axon terminal are important for maintaining neuronal function [22,23]. Previously, our laboratory demonstrated that blocking of axonal flow by colchicine administration and then accumulation of intracellular free zinc caused dentate granule cell death [24]. Recently, an interesting study demonstrated that genetic deletion of zinc transporter 3 (*ZnT3*), a putative transporter of zinc into synaptic vesicles, increases free zinc levels in the cytosol of neurons [25]. In addition, numerous studies have argued that Zn^2+^ has antioxidant properties in most systems, indicating a positive correlation between Zn^2+^ and GSH content [26,27,28,29]. Parat et al. reported that these antioxidant properties can be related to various actions, the most commonly described being Zn^2+^ interference with the absorption of other metals, such as Cu or Fe, and metal-catalyzed oxidation reactions, Zn^2+^ involvement in cooper/zinc superoxide dismutase (CuZnSOD) stability and the protection of thiol groups by Zn^2+^ [28].

In concert with the above findings, we propose the hypothesis that increased intracellular free zinc levels by *ZnT3* gene deletion may increase neuronal GSH levels, thereby preventing colchicine-induced oxidative injury in the dentate granule cell.

## 2. Results

### 2.1. Colchicine-Induced Dentate Granule Cell Degeneration Is Reduced in ZnT3^−/−^ Mice

**Figure 1:**
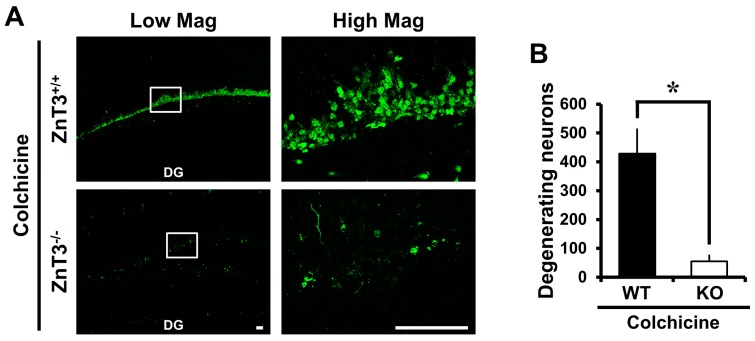

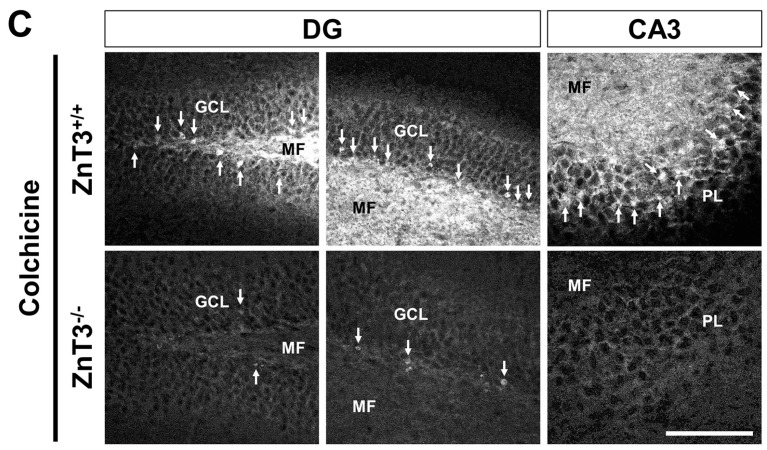
*ZnT3*^−/−^ mice exhibit reduced dentate granule cell death after colchicine injection. Brain sections obtained from WT and *ZnT3*^−/−^ (KO) mice at 24 h after colchicine injection (WT-colchicine, *n* = 4; KO-colchicine, *n* = 5) were analyzed by Fluoro-Jade B (FJB) staining to measure the degree of neurodegeneration. Representative images (**A**) and quantification (**B**) for the degree of neurodegeneration are shown as the number of degenerating cells of dentate gyrus (DG). Scale bar = 50 μm. Data are the mean ± SEM, * *p* < 0.05 versus WT mice; (**C**) Fluorescence photomicrographs show zinc-specific TSQ staining in the hippocampal DG and cornus ammonis 3 (CA3) at 24 h after colchicine injection. The dark holes represent the normal appearance of cell bodies, and the bright white fluorescence in the cell bodies (marked by a white arrow) indicates abnormal zinc accumulation. Scale bar = 100 μm. MF: mossy fiber. GCL: granular cell layer. PL: pyramidal cell layer.

It has previously been shown that the dentate granule cell is particularly vulnerable to colchicine and that this may contribute to subsequent cognitive impairment [30,31]. We first investigated whether genetic deletion of *ZnT3* influenced dentate granule cell death at 24 h after colchicine injection. Colchicine-induced dentate granule cell degeneration was analyzed by Fluoro-Jade B (FJB) staining to detect dying cells. Sham-operated groups didn’t show any FJB (+) cells. Intrahippocampal colchicine injection induced several FJB (+) cells in the dentate gyrus (DG). However, we found that *ZnT3*^−/−^ (KO) mice had a significantly reduced number of FJB (+) cells in the DG after colchicine injection, compared to the colchicine-injected WT mice (WT, 428.3 ± 84.06; KO, 55.5 ± 19.93; an 87% reduction). These results indicated that the lack of *ZnT3* suppressed the colchicine-induced dentate granule cell degeneration (Figure 1A,B).

### 2.2. ZnT3 Gene Deletion Prevents Intracellular Zinc Accumulation in the Dentate Granule Cells after Colchicine Injection

Next, we assessed if the *ZnT3* gene deletion can prevent intracellular zinc accumulation in the granule cells of DG after colchicine injection. Colchicine-induced intracellular zinc accumulation was detected by the zinc-specific stain TSQ. Hippocampal sections harvested 24 h after colchicine injection showed an intense fluorescence signal in the cell bodies of dentate granule cells, indicative of labile zinc accumulation in these cells. However, the number of TSQ (+) neurons in the DG and cornus ammonis 3 (CA3) was significantly reduced in *ZnT3*^−/−^ mice, compared to the colchicine-injected WT mice, indicating that deletion of the *ZnT3* gene prevented colchicine-induced intracellular zinc accumulation (Figure 1C).

### 2.3. ZnT3 Gene Deletion Showed Less Oxidative Injury after Colchicine Injection

**Figure 2:**
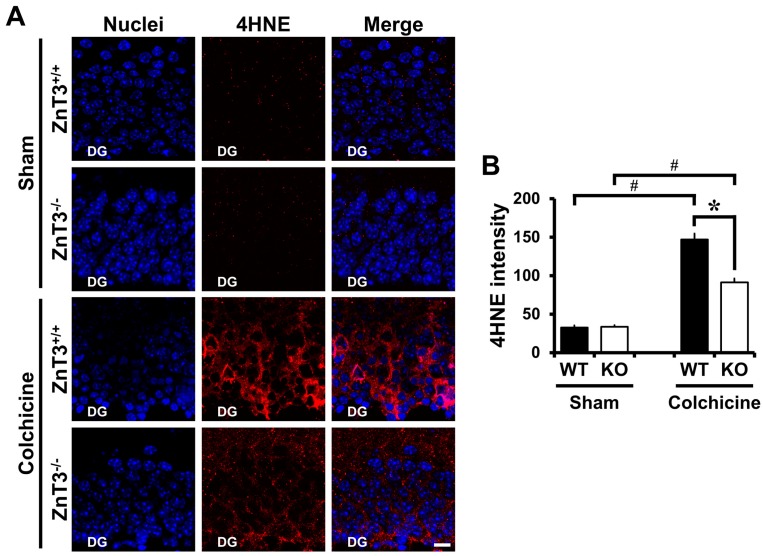
Colchicine-induced oxidative injury is reduced in *ZnT3*^−/^^−^ mice. WT and *ZnT3*^−/−^ mice were either sham-operated (WT-sham, *n* = 3; KO-sham, *n* = 3) or colchicine-injected (WT-colchicine, *n* = 4; KO-colchicine, *n* = 5). Brain sections were immunohistochemically stained with anti-4-hydroxynonenal (4HNE) to detect oxidative injury. (**A**) Representative images reveal 4HNE-labeled cells in the hippocampal DG from either WT or *ZnT3*^−/−^ mice at 12 h after sham surgery or colchicine injection. Scale bar = 10 μm; (**B**) The bar graph shows the intensity of 4HNE-immunoreactivity (IR) in the granule cell of DG from sham-operated and colchicine-injected mice of either WT or *ZnT3*^−/−^. Data are the mean ± SEM, * *p* < 0.05 versus WT mice, ^#^ *p* < 0.05 versus sham-operated mice.

We next examined whether reduced neuronal death after colchicine injection in mice lacking the *ZnT3* was related to less oxidative injury in the DG. To assess oxidative injury, brain sections were immunohistochemically stained with 4HNE at 12 h after colchicine injection. One of the major generators of oxidative stress, 4-hydroxynonenal (4HNE), has been widely considered as a bioactive marker of lipid peroxidation [32,33]. There were almost no 4HNE-stained granule cells in sham-operated mice of either WT or *ZnT3*^−/−^ (WT, 32.5 ± 2.77; KO, 33.6 ± 1.99; average gray scale intensities). The intensity of 4HNE-immunoreactivity (IR) was remarkably increased in DG of WT mice after colchicine injection. However, colchicine-injected *ZnT3*^−/−^ mice showed markedly less intensity of 4HNE-IR in the granule cell of DG, compared to the colchicine-injected WT mice (WT, 147.1 ± 7.49; KO, 91.4 ± 4.75; average gray scale intensities, a 38% reduction). These results suggested that the resistance of *ZnT3*^−/−^ mice to colchicine-induced dentate granule cell degeneration might be related to the reduced oxidative injury in the DG of hippocampus (Figure 2).

### 2.4. ZnT3 Gene Deletion Reduced Dendritic Damage after Colchicine Injection

**Figure 3:**
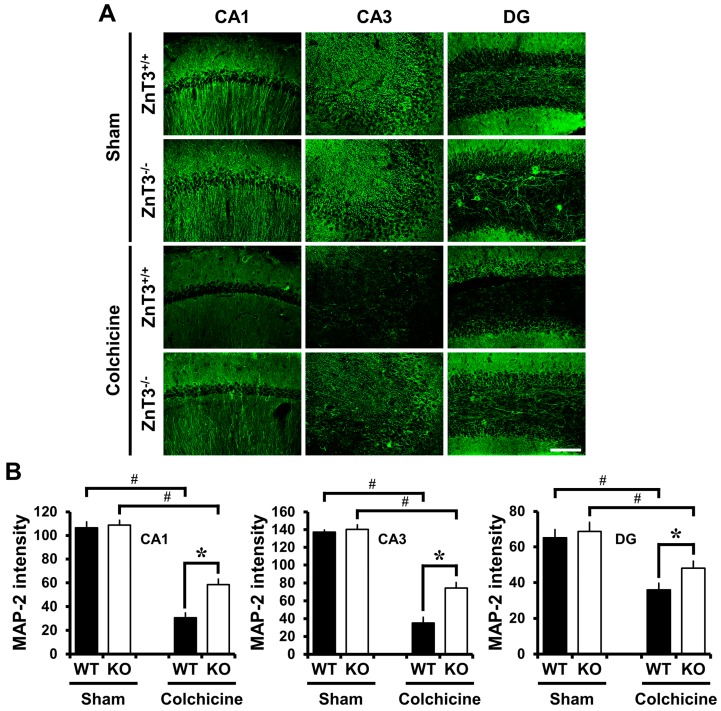
Genetic deletion of *ZnT3* reduces colchicine-induced dendritic damage. WT and *ZnT3*^−/−^ mice were either sham-operated (WT-sham, *n* = 3; KO-sham, *n* = 3) or colchicine-injected (WT-colchicine, *n* = 4; KO-colchicine, *n* = 5). Brain sections were immunohistochemically stained with 4HNE to detect oxidative injury. (**A**) Representative images reveal MAP2-IR in the hippocampal CA1, CA3 and DG from either WT or *ZnT3*^−/−^ mice at 24 h after sham surgery or colchicine injection. Scale bar = 100 μm; (**B**) The graph represents the intensity of MAP2-IR in the CA1, CA3 and DG from sham-operated and colchicine-injected mice of either WT or *ZnT3*^−/−^. Data are the mean ± SEM, * *p* < 0.05 versus WT mice, ^#^ *p* < 0.05 versus sham-operated mice.

We also determined whether deletion of *ZnT3* reversed colchicine-induced dendritic damage in the hippocampus. MAP2 is exclusively expressed by dendrites of neurons where it binds to tubulin [34] and is thought to be involved in microtubule assembly, acting to stabilize microtubules [35]. It is considered that the loss of MAP2 protein is a characteristic of dendritic damage [36]. There were no significant differences in the distribution of MAP2-IR between the sham-operated WT and *ZnT3*^−/−^ mice (cornus ammonis 1 (CA1): WT, 106.5 ± 5.10; KO, 108.9 ± 3.99; cornus ammonis 3 (CA3): WT, 137.3 ± 2.22; KO, 140.3 ± 5.07; DG: WT, 65.2 ± 4.54; KO, 68.7 ± 5.11; average gray scale intensities). In both cases, MAP2-IR was observed in the apical dendrites of the CA1 and CA3, as well as the dendrites of granule cells within the DG. Compared with the sham-operated group, expression levels of MAP2 in the hippocampal CA1, CA3 and DG were significantly reduced in WT mice 24 h after colchicine injection. In contrast, colchicine-injected *ZnT3*^−/−^ mice revealed highly increased IR to MAP2, compared to the colchicine-injected WT mice (CA1: WT, 30.6 ± 4.03; KO, 58.6 ± 4.53, a 91% increase; CA3: WT, 35.2 ± 6.42; KO, 74.3 ± 6.25, a 111% increase; DG: WT, 35.9 ± 3.70; KO, 48.0 ± 4.02, a 34% increase; average gray scale intensities). These results suggested that the lack of *ZnT3* inhibited the colchicine-induced dendritic damage in the hippocampus (Figure 3).

### 2.5. Colchicine-Induced Neuronal GSH Depletion Is Prevented in ZnT3^−/−^ Mice

**Figure 4:**
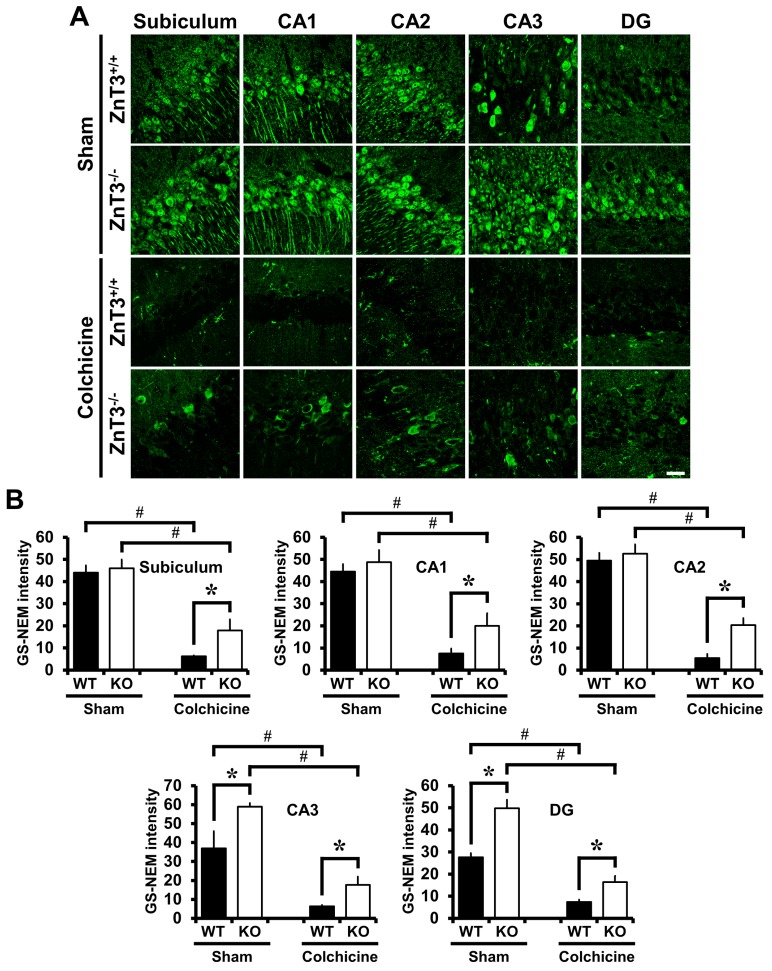
*ZnT3*^−/−^ mice have increased neuronal glutathione and reversed a reduction of neuronal glutathione (GSH) content after colchicine injection. Brain sections obtained from WT and *ZnT3*^−/−^ mice at 24 h after sham surgery or colchicine injection (WT-sham, *n* = 3; KO-sham, *n* = 3; WT-colchicine, *n* = 4; KO-colchicine, *n* = 5) were stained by GSH-N-ethylmaleimide (NEM) adduct (GS-NEM) antibody to detect the reduced form of GSH in the neurons. Representative immunofluorescence images (**A**) and quantification (**B**) for the degree of neuronal GSH levels are shown as the intensity of GS-NEM in the individual neurons of hippocampal regions including subiculum, CA1, CA2, CA3 and DG. Scale bar = 20 μm. Data are the mean ± SEM, * *p* < 0.05 versus WT mice, ^#^ *p* < 0.05 versus sham-operated mice.

To evaluate whether *ZnT3* gene deletion affected neuronal GSH, sections were histologically analyzed by probing for GSH-N-NEM adducts at 24 h after colchicine injection. Neurons in the hippocampal regions including subiculum, CA1, CA2, CA3 and DG were immunoreactive to the GS-NEM antibody, as can be seen in Figure 4A. GS-NEM IR in the subiculum, CA1 and CA2 of *ZnT3*^−/−^ mice was similar to that in the WT mice, but was higher in CA3 and DG (subiculum: WT, 44.0 ± 3.17; KO, 46.0 ± 4.00; CA1: WT, 44.5 ± 3.43; KO, 48.8 ± 5.39; CA2: WT, 49.4 ± 3.57; KO, 52.6 ± 4.17; CA3: WT, 36.9 ± 9.02; KO, 58.9 ± 1.83, a 60% increase; DG: WT, 27.6 ± 1.89; KO, 49.8 ± 3.73, a 81% increase). Colchicine injection caused a decrease in the level of GS-NEM IR in all hippocampal regions. In addition to that, *ZnT3*^−/−^ mice subjected to colchicine injection showed a significant increase of GS-NEM IR in all hippocampal regions, compared to colchicine-injected WT mice (subiculum: WT, 6.2 ± 0.35; KO, 17.9 ± 4.98, a 189% increase; CA1: WT, 7.5 ± 2.14; KO, 20.0 ± 5.66, a 167% increase; CA2: WT, 5.5 ± 1.81; KO, 20.4 ± 3.12, a 274% increase; CA3: WT, 6.3 ± 0.74; KO, 17.7 ± 4.31, a 181% increase; DG: WT, 7.4 ± 1.01; KO, 16.4 ± 2.68, a 123% increase). These results indicate that *ZnT3*^−/−^ mice have increased neuronal glutathione and decreased vulnerability to oxidants induced by colchicine injection (Figure 4).

## 3. Discussion

In the present study, we found that *ZnT3*^−/−^ mice exhibited reduced dentate granule cell death in the hippocampus after colchicine injection compared to WT mice. The reduction of colchicine-induced dentate granule cell death by *ZnT3* gene deletion is accompanied by suppression of oxidative injury, dendritic damage and zinc accumulation in the hippocampus. In addition, *ZnT3*^−/−^ mice showed a higher GSH level than WT mice in the hippocampus and showed reduced neuronal GSH depletion after colchicine injection. These findings suggest that the increased intracellular free zinc level by *ZnT3* gene deletion increased the neuronal GSH levels, thereby preventing the colchicine-induced dentate granule cell death.

Microtubules are one of the crucial cytoskeletal components in neurons, having an important role in stabilizing neuronal morphology because they provide platforms for intracellular transport that are involved in a variety of cellular cascades, including the movement of organelles, secretory vesicles and intracellular macromolecular assemblies, as well as controlling local signaling events [37]. Colchicine inhibits polymerization of microtubules by binding to β-tubulin [5], which is essential for cellular mitosis. It is also well-known to effectively act as a mitotic inhibitor used for cancer treatment. Since one of the characteristics of cancer cells is an increased rate of mitosis, cancer cells are more vulnerable to colchicine toxicity than normal cells. However, the therapeutic value of colchicine against cancer is limited by its toxicity against normal cells. Numerous studies indicated that colchicine causes the selective destruction of granule cells in the DG of the hippocampus [1,2]. In addition, our previous study has shown that cytoskeleton-disrupting agents such as colchicine or vincristine induce dentate granule cell death by blocking axonal zinc flow and intracellular zinc accumulation [24]. Therefore, these results suggest a new mechanism of neuronal death that arises by the blockade of the axonal zinc transport and subsequent intracellular zinc accumulation as intermediary steps in colchicine-induced dentate granule cell death.

It has been well established that the generation of free radicals and subsequent oxidative stress occur prior to neuronal death and play important roles in the pathogenesis of neurodegenerative diseases [15,16,17]. A previous study reported that central administration of colchicine is associated with an increase in free radical generation, and subsequent oxidative stress leads to cognitive dysfunction [3]. It has been reported that colchicine induces oxidative damage, possibly by the following mechanisms: (i) it causes an increase of the glutamate/γ-aminobutyric acid (GABA) ratio in the cortex of mice [38], and this relative increase in glutamate activity exhibits neurotoxic effects by generating hydroxyl free radicals [39]; (ii) colchicine also leads to increased production of nitric oxide (NO) and inducible nitric oxide synthase (iNOS) in the brain [40]; the generated NO can induce the peroxynitrite-mediated formation of free radicals by interacting with the superoxide anion; (iii) the generated NO also causes nitrosylation of diverse enzymes, thereby inhibiting glycolysis and inducing brain damage [41]. Our lab also demonstrated that NO increased vesicular zinc release and subsequent intracellular free zinc accumulation [42].

**Figure 5:**
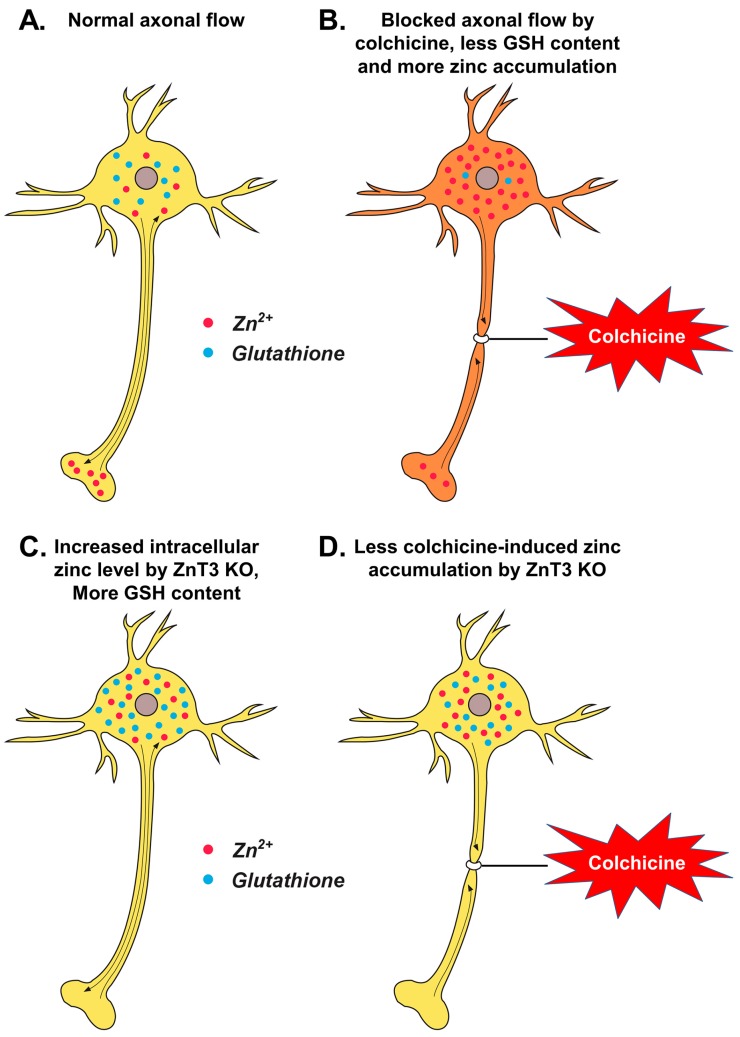
Proposed mechanism by which *ZnT3* knockout reduces colchicine-induced dentate granule cell degeneration. This schematic drawing indicates several chain reactions that are thought to occur after colchicine injection in *ZnT3*^−/−^ mice. (**A**) Cytoplasmic and vesicular zinc are normally moved by the axonal flow in WT mice. Zinc transport is essential for axonal flow in neurons. In addition, the concentration of GSH is much greater in the neuronal cytoplasm; (**B**) Blocked axonal flow by colchicine induces depletion of GSH content and an increase of intracellular zinc accumulation, thereby causing neuronal death; (**C**) *ZnT3* gene deletion causes an increased intracellular free zinc level and increased neuronal GSH levels; (**D**) Genetic deletion of *ZnT3* attenuates colchicine-induced zinc accumulation and dentate granule cell death.

In the present study, intrahippocampal injection of colchicine caused significant injury of dentate granule cells as evidenced by increased oxidative stress, dendritic damage, zinc accumulation and neuronal death. Furthermore, the colchicine-induced dentate granule cell death was significantly reduced in *ZnT3*^−/−^ mice. *ZnT3* is mainly localized at Zn^2+^-containing synaptic glutamatergic vesicles in the hippocampus, cortex and olfactory bulb. Yoo et al. have demonstrated that the free Zn^2+^ level is higher in neuronal somata of *ZnT3*^−/−^ mice than those of wild-type mice, and metallothioneins 1 and 2 (*Mt1/2*), which are known to be induced by increases in cytosolic free Zn^2+^ levels, were also substantially increased in *ZnT3*^−/−^ mice [25]. Glutathione is a tripeptide composed of glutamate, glycine and cysteine. It plays a major role as an endogenous antioxidant present in the reduced form within cells [12]. It has been shown to react with free radicals and prevents the generation of hydroxyl free radicals [14]. The decreased GSH level and glutathione S-transferase (GST) activity after colchicine injection suggest that there was an increase in free radical generation and a depletion of the GSH-dependent antioxidant system during oxidative stress. Several studies have argued that Zn^2+^ status affects glutathione concentrations in tissues [26,27,29,43]. Parat et al. demonstrated a positive correlation between intracellular zinc deprivation and GSH depletion in cells treated with the zinc chelator *NNN′N′*-tetrakis(2-pyridylmethyl)ethylenediamine (TPEN) [28]. In the present study, results showed that *ZnT3*^−/−^ mice had more reduced glutathione (GSH) content than WT mice in the hippocampal CA3 and DG. Furthermore, *ZnT3*^−/−^ mice showed prevention of colchicine-induced neuronal GSH depletion in the hippocampal subiculum, CA1, CA2, CA3 and DG. Although the present study suggests that *ZnT3* gene deletion causes an increased intracellular free zinc level and increased neuronal GSH levels, the exact mechanism of how *ZnT3* gene deletion provides neuroprotective effects is not clear. The present study is seemingly paradoxical insofar that *ZnT3*^−/−^ mice showed increased intracellular levels of zinc as presented in the previous study [25], but the proposed diagram in Figure 5 suggests that there is less accumulation of zinc. Thus, we hypothesize that this is in relation to the effect that the accumulation of zinc in response to colchicine is due to impaired axonal transport specifically at 24 h, whereas *ZnT3*^−/−^ mice might have alternative mechanisms for dealing with a normally elevated level of zinc.

Taken together, we suggest that the increased intracellular free zinc level by *ZnT3* gene deletion is correlated with increased neuronal GSH levels, which in part provide neuroprotective effects in the colchicine-induced oxidative injury in dentate granule cell.

## 4. Materials and Methods

### 4.1. Mouse Colonies

The animal care protocol and experimental procedures were approved by the committee on animal use for research and education at Hallym University (Protocol # Hallym 2014-28), in accordance with NIH guidelines. This manuscript was written in compliance with the guidelines of ARRIVE (animal research: reporting in vivo experiments) [44]. Wild-type (WT) and *ZnT3* KO male mice (background strains, C57BL/6 and Sv129 hybrid), aged 8 weeks, were propagated and maintained in the facility of Hallym University, College of Medicine. Mice were housed in a regulated environment (22 ± 2 °C, 55 ± 5% humidity, 12:12 h light:dark cycle with lights on at 8:00 a.m.) and received a standard diet by Purina (Purina, Gyeonggi, Korea). Food and water were accessed ad libitum. As described previously [45], PCR genotyping was performed with a primer set to amplify WT (5′-GGT ATC CAT GCC CTT CCT CTA GAG-3′), or common (5′-ATA GTC ACT GGC ATC CTC CTG TAC C-3′), or the KO allele (5′-CCT GTG CTC TAG TAG CTT TAC GG-3′) prior to all experiments. The WT band is 650 bp in size, and the KO band is 400 bp in size.

### 4.2. Colchicine Intrahippocampal Injection and Experimental Design

To assess the role of *ZnT3* on colchicine-induced dentate granule cell degeneration, colchicine (10 µg/kg; Sigma, St. Louis, MO, USA) was intrahippocampally injected as previously described [24]. Briefly, mice were deeply anesthetized with isoflurane (1–2% for maintenance; 3% for induction) in a 70:30 mixture of nitrous oxide and oxygen using an isoflurane vaporizer (VetEquip Inc., Livermore, CA, USA) and positioned in a stereotaxic apparatus (David-Kopf Instruments, Tujunga, CA, USA). A burr hole was made in the skull, and a 29-gauge needle was inserted through the burr hole into the hippocampus (coordinates: 1.4 mm lateral from the midline, 1.5 mm posterior to the bregma and 1.8 mm below the cortical surface). We then slowly injected colchicine and removed it after placement for another 5 min. The burr hole was sealed with bone wax. Following the suture of the skin incision, anesthetics were discontinued. When mice showed spontaneous respiration, they were returned to a recovery room maintained at 37 °C. Core temperature was kept at 36.5–37.5 °C with a homoeothermic blanket control unit (Harvard apparatus, Holliston, MA, USA). Sham-operated mice received the same skin incision under isoflurane anesthesia, but they were administered with 1 μL sterile saline into the hippocampus. Mice were divided into four groups for histological evaluation: (1) sham-operated WT mice (WT-sham, *n* = 6), (2) sham-operated *ZnT3*^−/−^ mice (KO-sham, *n* = 6), (3) colchicine-induced WT mice (WT-colchicine, *n* = 8) and (4) colchicine-induced *ZnT3*^−/−^ mice (KO-colchicine, *n* = 10).

### 4.3. Tissue Preparation

Mice were perfused with 0.9% saline followed by 4% paraformaldehyde (PFA) under urethane (1.5 g/kg, i.p.) anesthesia. The brains were post-fixed with 4% PFA in phosphate-buffered saline (PBS) for 1 h and then immersed in 30% sucrose until subsided for cryo-protection. Thereafter, the entire brain was frozen and coronally sectioned at a 30 μm thickness using a cryostat (CM1850, Leica, Wetzlar, Germany).

### 4.4. Detection of Cell Death

Cell death after colchicine injection was evaluated after a 24 h survival period. To identify degenerating cells, Fluoro-Jade B (FJB, Histo-Chem, Jefferson, AR, USA) staining was used [46]. Five coronal sections (180 µm intervals, collected from 1.2–2.1 mm caudal to bregma) were analyzed from each mouse. A blinded experimenter counted the total number of FJB-positive cells in the hippocampal dentate gyrus (DG) from the ipsilateral hemisphere. Data were represented as the average number of degenerating cells per each region.

### 4.5. Detection of Neuronal Glutathione (GSH)

To detect the reduced form of GSH in the brain sections, we probed for GSH-*N*-ethylmaleimide (NEM) adducts on the free-floating coronal sections [47,48,49]. Brain sections were incubated with 10 mM NEM for 4 h at 4 °C, washed and incubated with mouse anti-GS-NEM (diluted 1:100, Millipore, Billerica, MA, USA). After washing, the sections were incubated with Alexa Fluor 488-conjugated goat anti-mouse IgG (diluted 1:250, Invitrogen, Carlsbad, CA, USA) for 2 h. The sections were mounted on gelatin-coated slides, and fluorescence signals were detected using a Zeiss LSM 710 confocal imaging system (Carl Zeiss, Oberkochen, Germany). Stacks of images (1024 × 1024 pixels) from consecutive slices of 0.9–1.2 μm in thickness were obtained by averaging eight scans per slice and were processed with ZEN 2010 (Carl Zeiss, Oberkochen, Germany). To quantify GSH intensity, individual neurons from the brain section images were selected as regions of interest (ROIs) and measured using ImageJ (NIH, Bethesda, MA, USA). Briefly, to quantify the GSH intensity, the image was loaded into ImageJ v. 1.50f and converted into an 8-bit image through the menu option Image → Type → 8-bit. Then, regions comprising individual neurons in the subiculum, CA1, CA2, CA3 and DG images were selected as ROIs. The resulting image was then binarized and restricted to the region of measurement for individual neurons. To measure this area, the menu option Analyze → Measure was selected, and then the signal from individual neurons was expressed as the mean gray value [49,50,51].

### 4.6. Immunofluorescence Staining

The immunolabeling procedures were performed as per routine immunostaining protocols. To block endogenous peroxidase activity, sections were immersed in 3% hydrogen peroxide for 15 min at room temperature. After washing in PBS, the sections were incubated with the following antibodies as primary antibodies: a rabbit polyclonal anti-4-hydroxynonenal (4HNE, recognizing oxidative stress, diluted 1:500, Alpha Diagnostic International, San Antonio, TX, USA) or a mouse monoclonal anti-microtubule-associated protein 2 (MAP2, recognizing a neuron-specific cytoskeletal protein, diluted 1:200, Millipore, Billerica, MA, USA). For visualization of antibody binding, Alexa Fluor 488- and 594-conjugated antibodies were applied at a dilution of 1:250 as secondary antibodies. Between incubations, the sections were washed with PBS 3 times for 10 min each and then mounted on gelatin-coated slides. According to the method modified from the above-described method, 4HNE or MAP2 intensity was quantified using ImageJ. The image was converted to 8 bit through the menu options (Image/Type/8-bit). Next, the image was thresholded as follows (Image/Adjust/Threshold): the type was set to black and white and the bottom slider moved to a value sufficient to show only the 4HNE or MAP2 immunoreactive area. The thresholded image was binary and only represented 4HNE or MAP2 immunoreactivity. The selected part in the whole image was sorted, and then the intensity of 4HNE or MAP2 was expressed as the mean gray value.

### 4.7. Free Zinc Staining

For analyzing the vesicular and intraneuronal free zinc, the fresh frozen brain sections were stained with *N*-(6-methoxy-8-quinolyl)-para-toluenesulfonamide (TSQ, Molecular Probes, Eugene, OR, USA) fluorescent probes as previously described [52]. TSQ is a membrane-permeant Zn^2+^ indicator. Mice were anesthetized with 3–5% isoflurane in oxygen. Brain tissue was harvested without perfusion, quickly frozen on powdered dry ice and then stored at −80 °C. Unfixed fresh frozen brains were coronally sectioned with a 25 μm thickness. Sections were immersed for 1 min in a solution of 4.5 μM TSQ, 140 mM sodium barbital and 140 mM sodium acetate (pH 10.5–11), then rinsed for 1 min in 0.9% normal saline. TSQ–zinc binding was evaluated using an Olympus upright fluorescence microscope with an excitation/emission wavelength of 360/490 nm and photographed using a charge-coupled device (CCD) cooled digital color camera (Hamamatsu Co., Bridgewater, NJ, USA) with Infinity 3 (Lumenera Co., Ottawa, ON, Canada).

### 4.8. Statistical Analysis

Comparisons between experimental groups were conducted using repeated measures analysis of variance (ANOVA) followed by the Student–Newman–Keuls post hoc test. Data were presented as the mean ± SEM, and differences were considered significant at *p* < 0.05.
